# The Acute Effect of Increasing Resistance Training Workload Volume on Muscle Damage Markers and Performance in Heavy Resistance-Trained Youth Athletes

**DOI:** 10.3390/sports14040142

**Published:** 2026-04-03

**Authors:** Liam Bartlett, Anthony Scott Leicht, Wade Heath Sinclair, Jonathan Douglas Connor, Kenji Doma

**Affiliations:** College of Healthcare Sciences, James Cook University, Townsville, QLD 4814, Australia; liam.bartlett@my.jcu.edu.au (L.B.); anthony.leicht@jcu.edu.au (A.S.L.); wade.sinclair@jcu.edu.au (W.H.S.); jonathan.connor@jcu.edu.au (J.D.C.)

**Keywords:** creatine kinase, muscle soreness, physiological adaptation, vertical jump, sprint

## Abstract

Despite the widespread use of periodized resistance training by athletes, the acute physiological and performance responses when athletes transition between mesocycles with differing workload volumes remain poorly understood. This study examined the acute effect of increasing resistance training workload volume on muscle damage markers and field-specific performance in heavy resistance-trained youth athletes. Eighteen male, rugby league players (age 17.4 ± 0.8 years; body mass 80.2 ± 13.7 kg; height 1.8 ± 0.1 m) completed a four-week mesocycle to develop maximal strength (70–100% of one repetition maximum [1RM]). Muscle damage (i.e., delayed onset muscle soreness [DOMS] and creatine kinase [CK]) and performance measures (i.e., drop jump, plyometric push-up, 40 m sprint and repeated agility) were assessed prior to and at 24 h (T24) and 48 h (T48) following the last session of the strength mesocycle (Week 5). A hypertrophy session (35–70% of 1RM) was then included in Week 6 with data collected prior to and at T24 and T48. Compared with the strength (Week 5) modality, the hypertrophy (Week 6) modality resulted in greater DOMS (41.6 ± 22.7%; effect size [ES] = 0.97–1.12) and modestly higher CK (26.7 ± 47.8%; ES = 0.6). Larger declines in field-specific performance measures were also shown during the HYP modality than STR modality for 20 m sprint performance (−2.1 ± 4.3%; ES = 0.7) and agility performance (−1.1 ± 4.2%; ES = 0.6). There were more modest reductions in drop jump performance (−4.1 ± 7.2%; ES = 0.7) during the HYP modality than in the STR modality, although caution should be given as two separate force plate systems were combined due to technical difficulties. Increasing workload volume was associated with greater muscle damage and modest differences in selected field-specific performance measures following several weeks of maximal strength training. These findings provide preliminary insight into the acute responses to increases in resistance training workload volume. Coaches should monitor athletes’ acute responses during fluctuations in workload volume and consider strategies to help maintain training quality in youth athletes.

## 1. Introduction

Rugby league is an intense, collision sport that requires lean muscle mass, muscular strength and power, agility, speed, and repeated sprint ability [1,2]. Thus, rugby league players commonly undertake strenuous resistance training to meet the demands of their sport [3,4]. Resistance training for competitive athletes, including rugby league players, is typically prescribed in a periodized format, consisting of various training phases within and outside of the competition season [5]. These training phases are referred to as mesocycles, with each lasting 2–6 weeks and inclusive of a distinct training focus that supplies sufficient training stimuli for muscular adaptations [6].

During the preseason, athletes typically commence periodized resistance training with moderate loads (70–85% of 1-repetition maximum, RM) and higher volume (3–4 sets ×6–10 repetitions) to develop hypertrophy. As athletes regain muscular strength within the early pre-season, their resistance training transitions to focus on maximum strength, consisting of moderate volume (3–6 sets × 2–6 repetitions) and heavy loads (85–100% of 1RM) [7,8]. Given the intense nature of the sport, mesocycles that focus on developing strength are crucial for rugby league athletes [9]. However, constant heavy-loading resistance training appears to only increase, or maintain, muscular strength and power performance for approximately 4–5 weeks [10]. Any longer resistance training periods at such high intensity may cause monotonous program overtraining, resulting in loss or plateauing of performance [11]. As such, rugby league players fluctuate their workload volume [12], typically calculated as sets × repetitions × weight lifted, and may reintroduce higher volume, lighter load resistance training following a period of maximal strength training in a wavelike progression [13]. Thus, training load and volume are altered as the periodization model transitions across each mesocycle. Whilst a heightened training response is desirable to overcome training plateaus and enhance chronic adaptation, athletes may initially experience high levels of exercise-induced muscle damage (EIMD), given that fluctuations in workload volume expose athletes to an unfamiliar training stimulus [14].

Common symptoms of EIMD include increased muscle soreness (i.e., delayed onset muscle soreness, DOMS) and intra-muscular proteins (e.g., creatine kinase, CK), and a temporary loss in muscle functional capacity that can last for several days [15,16]. Schoenfeld [17] suggested that EIMD can be amplified when the workload volume of resistance training is increased. In fact, Abboud et al. [18], reported greater EIMD markers (i.e., CK and DOMS) during a higher workload volume week (20,000 kg vs. 10,000 kg per week), suggesting a higher resistance training workload volume produces greater EIMD. However, Abboud, Greer [18], examined alterations in workload volume across two resistance training sessions, rather than in a periodized context involving multiple resistance training sessions. A systematic review and meta-analysis [19] partly confirmed this link between workload volume and EIMD in a periodized context, with increased levels of EIMD reported when training loads were increased during various times of a sporting season in competitive athletes. However, the systematic review [19] considered workload volume based on a variety of exercises, and acute responses to changes in workload volume were not compared across successive weeks. Whilst previous studies suggest that an acute increase in workload volume elevates the level of EIMD, it is important to consider that prior exposure to resistance training elicits lower levels of EIMD due to the repeated bout effect [20]. Given that competitive rugby league athletes consistently undertake resistance training, alterations in workload volume may limit changes in the level of EIMD experienced by athletes.

Coutts, Reaburn [21], explored the acute effects following a mesocycle of resistance and field-based training in semi-professional rugby league players. The authors [21] prescribed an intensified, six-week mesocycle with limited recovery to deliberately induce overreaching. At the conclusion of the mesocycle, most field and laboratory-based performance measures were decreased with a concomitant increase in CK. These findings indicated that an intensified mesocycle impaired performance in trained rugby league players, possibly due to elevated EIMD based on the level of CK. Although these findings highlighted the effects of an intensified mesocycle, such a training method is employed sparingly due to the increased likelihood of overtraining [21]. Commonly, training for rugby league athletes consists of cyclic variations in resistance training workload volume in a periodized manner, with workload volume potentially increased following a mesocycle of heavy-loaded resistance training with lower workload volume [13]. The effect of such cyclic transitions on acute physiological and performance measures of rugby league athletes though remains unknown. Developing a greater understanding of the acute responses to changes in resistance training workload volume during periodization could assist practitioners with their management of EIMD symptoms, training quality and minimization of injury risks [22]. Therefore, the aim of this study was to examine the effect of transitioning between different mesocycles by altering workload volume on indirect muscle damage markers and field-specific performance measures in resistance-trained youth rugby league players. It was hypothesized that players would exhibit a greater level of EIMD with impairment in explosive-strength and field-specific performance measures, including sprint and agility performance, when transitioning from a strength to a hypertrophy mesocycle. This hypothesis was derived on the basis of the repeated bout effect [20], where the players would develop tolerance to maximal strength training-induced stress during the initial four weeks of training, whilst experiencing heightened resistance training-induced stress due to the unfamiliarity with hypertrophy training.

## 2. Materials and Methods

### 2.1. Participants

Eighteen adolescent males (age 17.4 ± 0.8 yrs; body mass 80.2 ± 13.7 kg; height 1.8 ± 0.1 m) from local rugby league sporting teams volunteered to participate in this study. The inclusion criteria were participants with at least two years of resistance training experience and four years of experience playing rugby league. The exclusion criteria were those that were over 18 years old and those that were injured in the previous 6 months. Participants wore the same footwear for all sessions; refrained from caffeine ingestion within two hours prior to each session; refrained from supplementation that would aid recovery; and maintained sleep and dietary habits and avoided additional strenuous physical activity. Participants also completed all exercise and testing protocols at the same time of day at the same venue. Participants were required to complete at least 80% of the four-week maximal strength training mesocycle to ensure adequate exposure to the training stimulus prior to the experimental sessions. This criterion applied only to the preparatory training period and not to the experimental testing sessions. All participants who met the attendance requirement subsequently completed both the final maximal strength session (Week 5) and the hypertrophy session (Week 6), and no outcome measurements were missing during these sessions. The testing and training sessions were completed following approval by the local institutional ethical committees. This study was approved by the Institutional Human Research Ethics Committee and conducted in accordance with the Declaration of Helsinki. The participants, as minors, and their parents/guardians were informed of the benefits and health risks of the study prior to providing written informed consent (ethics approval number H7861). According to an a priori sample size calculation from previous studies [23] for CK, DOMS, sprint and jump performance measures with expected effect sizes of 0.77, 0.43, 0.37 and 0.26, respectively, and expected correlation between repeated measures of at least 0.7, 14 participants was sufficient to detect a significant change (alpha level at 0.05 and 80% power; G*Power 3.1.9.2, Heinrich-Heine-Universität, Düsseldorf, Germany).

### 2.2. Research Design

**Figure 1:**
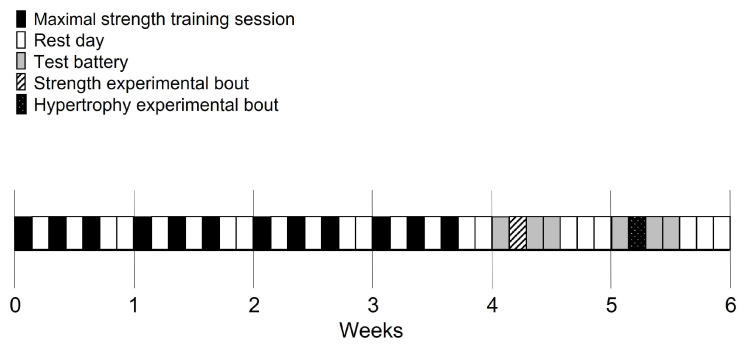
Schematic of research design. The test battery consisted of drop jump, plyometric push-ups, sprint and repeated sprint T-agility.

This study was conducted across six-weeks with participants completing a four-week mesocycle to develop maximal strength during the first four weeks (70–100% of one repetition maximum [1RM] with 4 sets of 1–5 repetitions), an additional maximal strength exercise session (STR; 70–95% of 1RM with 4 sets of 1–3 repetitions) in week five and a hypertrophy exercise session (HYP; 35–70% of 1RM with 3 sets of 8–12 repetitions) in week six (Figure 1). The training session consisted of four supersets throughout the program and the exercise order was maintained as outlined in Table 1. Within each superset, the primary exercises were performed first (e.g., barbell back squat in Table 1: 1a before the secondary movement of single leg calf raise in Table 1: 1b). The participants were required to complete each superset within 3 min, allowing approximately 1 min to complete each superset with 2 min of recovery. The RM test was conducted prior to, and at the conclusion of the four-week maximal strength mesocycle. During this period, participants completed twelve resistance training bouts as well as familiarization sessions for the testing battery. A battery of tests was conducted at baseline (i.e., 24 h before the STR and HYPs) and again at 24 h (T24) and 48 h (T48) following each session. The testing battery for EIMD consisted of CK and DOMS whilst completing a squat (DOMS-SQ) and a push up (DOMS-PU) using a visual analog scale from 1 to 10 (i.e., 1–2 = “not sore”; 3–4 = “Somewhat sore”; 5–6 = “Sore”; 7–8 = “Very sore”; 9–10 = “Very very sore”) [20]. Furthermore, performance measures included lower limb explosive-strength (drop box jump), upper body explosive-strength (plyometric push up), speed (40 m linear sprint) and repeated-agility (repeated T-test), key fitness components for by rugby league athletes [3]. Participants provided a 1–10 rating of perceived exertion (RPE) score for each exercise during the strength and hypertrophy bouts, with 1 classified as “very light” and 10 as “maximal effort”. The participants were provided with 48 h of passive rest prior to the battery of tests at baseline and were requested to avoid any form of strenuous activity during this time. The participants were also requested to maintain their dietary habits during the study.

### 2.3. Creatine Kinase

Creatine kinase levels based on isoform muscle–muscle (MM) were measured using a colorimetric assay procedure (Reflotron: Boehringer Mannheim, Mannheim, Germany). Using a 30 microlitre fingertip, capillary blood was pipetted immediately onto a test strip. The intra-assay coefficient of variation for the current CK assay procedure using the same equipment was previously reported as 7.2%, inter-day, intra-class correlation coefficient as 0.72, and minimal detectable change as 190.9 U∙L^−1^ [20].

#### Drop Box Jump

Participants completed a drop jump protocol to assess lower-body explosive-strength by stepping off a 30 cm box, landing on the force plate and jumping immediately as high as possible [20]. Due to technical difficulty, jump metrics were recorded using the AMTI force plate (Watertown, MA, USA) for the first 11 participants, whilst a separate force plate (ForceDecks, Jump Application v2.07782; Vald Performance, Brisbane, Qld, Australia) was used for the subsequent 7 participants. Due to further technical difficulty, the baseline measures for the STR using the ForceDecks were not available. Subsequently, baseline measures from the ForceDecks during the HYP were substituted for the STR to enable analysis. A 3 min dynamic warm-up (body weight squats, lunges and leg swings) was conducted by participants before their completion of three drop box jumps with each interspersed by 20 s of passive recovery [20]. Ground contact time and flight time (drop jump flight time, [DJ-FT]) were recorded in seconds, while the reactive strength index (RSI) was calculated by dividing flight time by ground contact time [24].

### 2.4. Plyometric Push up

After a dynamic warm-up (push-ups and arm swings), participants completed a plyometric push-up test on a force plate, by lowering their chest to the force plate before extending their arms and pushing their body up explosively. The participants completed three trials, each interspersed by 20 s of passive recovery and the greatest plyometric push-up flight time (PP-FT) recorded for later analyses. The PP-FT was recorded using an AMTI force plate (Watertown, MA, USA) for the first 11 participants, whilst a separate force plate (ForceDecks, Jump Application v2.07782; Vald Performance, Brisbane, QLD, Australia) was used for the subsequent 7 participants (including substitution of baseline results for the STR) [25].

### 2.5. Sprint

Following a dynamic warm up, participants completed three, 40 m sprints with each interspersed by a two-minute, passive recovery period. Participants commenced each sprint from 0.5 m behind the starting line to nullify any reaction time and anticipation effect [23]. Sprint, flying start, and acceleration times of 0–10 m, 0–20 m, 0–40 m, 10–20 m, 10–40 m and 20–40 m were recorded using timing gates (Speedlight Timing Gates, Swift Performance, Wacol, QLD, Australia) that were positioned at 0 m, 10 m, 20 m and 40 m.

### 2.6. Repeated T-Agility Test

**Figure 2:**
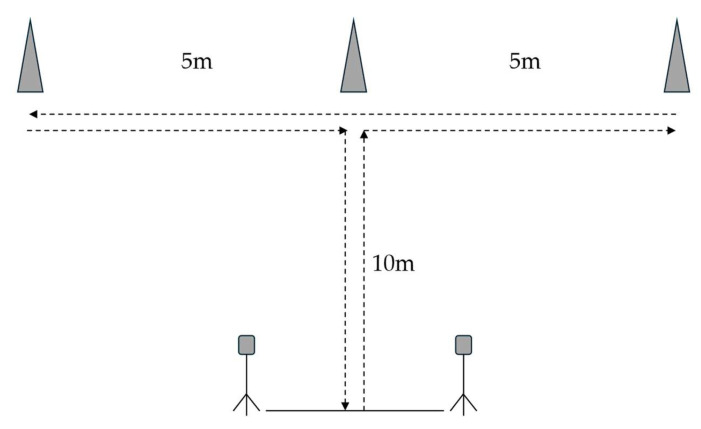
Schematic of the repeated T-test agility protocol. The dotted arrows indicate the direction of sprint.

Participants completed a repeated T-test agility protocol consisting of six trials, with each separated by 45 s based on a previous described protocol [26] (Figure 2). Prior to the protocol, participants completed a single-effort T-test trial at 50% effort followed by 60 s of passive recovery, and then another single-effort T-test trial at 100% effort as a warm-up. Participants received verbal encouragement during each trial while timing gates (Speedlight Timing Gates, Swift Performance, Wacol, QLD, Australia) were used to record the completion time of each trial and RPE recorded after each trial. The best time, average time, total time, fatigue index ((worst time—best)/best time × 100), average RPE, and peak RPE were recorded for the protocol. The repeated T-test protocol was previously reported to exhibit acceptable validity (>0.90) and exceptional reliability (ICC ≥ 0.97) for junior rugby league athletes [26].

### 2.7. Statistical Analysis

All data was reported as mean and standard deviation, and analyzed using the Statistical Package for Social Sciences (IBM SPSS, version 25; IBM Corp., Armonk, NY, USA). A two-way (exercise modality × time) repeated measures analysis of variance (ANOVA) compared indirect markers of muscle damage, explosive-strength, and field-specific performance measures between modalities (strength and hypertrophy sessions) and time points (i.e., baseline, T24 and T48) separately for each outcome measure. Effect sizes for the ANOVA effects were reported as partial eta-squared (*ηp*^2^) values obtained from the repeated-measures ANOVA output in SPSS. Mauchly’s test was used to assess the assumption of sphericity for repeated measures factors. When the assumption of sphericity was violated, Greenhouse–Geisser corrections were applied to adjust the degrees of freedom. Post hoc analyses were conducted using pairwise comparisons with a Bonferroni correction. Paired T-tests were used to compare muscular strength and RPE values. The data was tested for normality using the Shapiro–Wilk’s test, with log-transformation applied to outcome measures that were departed from normalized data. Percentage changes in outcome variables were also calculated for each participant from baseline to T24 and baseline to T48. The mean and standard deviation of these individual percentage changes were then calculated for each modality (strength and hypertrophy) at each time point. To determine the magnitude of differences between modalities, effect sizes were calculated by comparing the percentage changes between the strength and hypertrophy modalities at each time point. Effect sizes were calculated using Hedges’ *g* for repeated-measures designs, which accounts for the within-subject correlation between paired observations. Effect sizes were interpreted according to conventional thresholds of small, moderate, and large effects with values pertaining to 0.2, 0.5 and 0.8, respectively [27]. A sensitivity analysis was conducted to examine the potential influence of using different force plate systems by repeating the two-way ANOVA and effect size calculations for athletes that were tested on each force plate system. Statistical significance was set at *p* ≤ 0.05 for all analyses.

## 3. Results

### 3.1. Muscular Strength Measures

**Figure 3:**
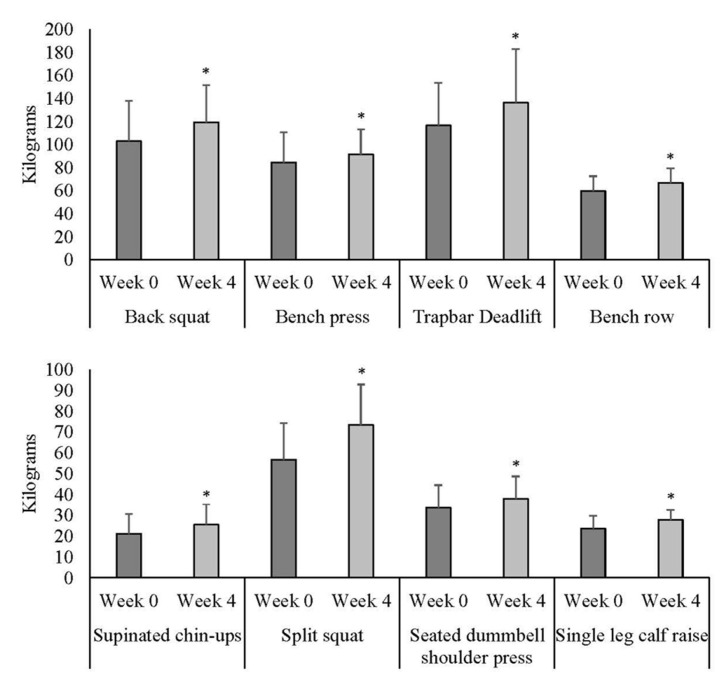
Mean ± standard deviation (error bars) of the mass lifted during repetition maximum testing for each exercise at the initial (Week 0) and end (Week 4) of the 4-week maximal strength training program (*n* = 18). * Significant differences between Week 0 and Week 4 (*p* < 0.05).

Based on normality assumptions, the raw scores for the 1RM results were used. According to the analyses, all training exercises were significantly increased from the start (Week 0) to the conclusion of the four-week strength program (Week 4, Figure 3).

### 3.2. Rating of Perceived Exertion

**Figure 4:**
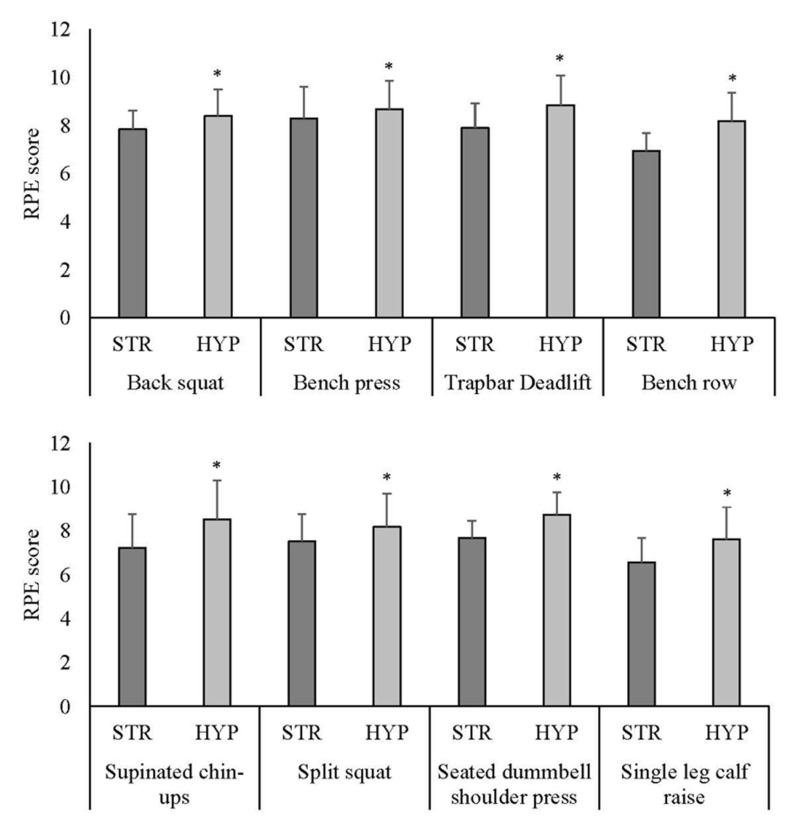
Mean ± standard deviation (error bars) of rating of perceived exertion (RPE) after each resistance exercise during the maximal strength exercise session (STR) in Week 5 and the hypertrophy exercise session (HYP) in Week 6 (*n* = 18). * Significant differences between STR modality and HYP modality (*p* < 0.05).

Based on normality assumptions, the raw scores for the RPE measures were used. During the exercise sessions, RPE was significantly greater for all activities within the hypertrophy modality when compared to the strength modality (Figure 4).

### 3.3. Muscle Damage Markers

Based on normality assumptions, the raw scores for DOMS were used for analyses, whilst CK was log-transformed with spaghetti plots of back-transformed data provided as Supplementary Files. There was an interaction effect for DOMS-SQ (F(2,34) = 45.6; *p* < 0.001; *ηp*^2^ = 0.73) and DOMS-PU (F(2,34) = 18.6; *p* < 0.001; *ηp*^2^ = 0.52), with significantly higher measures at T24 (*p* < 0.001) and T48 (*p* < 0.001) when compared to baseline for the hypertrophy modality (Table 2). Additionally, DOMS-SQ and DOMS-PU at T24 (*p* < 0.001) and T48 (*p* < 0.001) were significantly higher for the hypertrophy modality when compared to the strength modality (Table 2). Effect size calculations showed larger measures of DOMS-SQ and DOMS-PU for the hypertrophy modality than strength modality at T24 and T48 with large effects. There was also an interaction effect for CK (F(2,34) = 4.0; *p* = 0.027; *ηp*^2^ = 0.19), with significantly greater CK at T24 and T48 when compared to baseline for both strength and hypertrophy modalities (*p* > 0.01). Furthermore, CK was significantly greater after the hypertrophy modality than strength modality at T24 (*p* < 0.01) and T48 (*p* = 0.048). Effect size calculations also showed a moderate effect size between modalities at T24.

### 3.4. Explosive-Strength Measures

Raw data was used for all the explosive strength measures as the data was normally distributed. An interaction effect was identified for DJ-FT (F(2,34) = 4.4; *p* = 0.02; *ηp*^2^ = 0.20) with significantly lower measures for the hypertrophy modality compared to the strength modality at T24 (*p* = 0.03) and T48 (*p* = 0.02) (Table 2). Furthermore, DJ-FT was significantly lower at T24 (*p* = 0.02) and T48 (*p* = 0.004) when compared to baseline during the hypertrophy modality. No interaction effects or main effects of time or modality were identified for the other explosive-strength measures (*p* > 0.05; Table 2). The effect size calculations showed small differences between modalities at T24 and T48 for most of the explosive-strength measures, except DJ-FT was poorer for the hypertrophy modality than the strength modality with moderate effects at T24 and T48. According to the sensitivity analyses, the DJ-FT for the 11 participants that performed the jump protocol with the AMTI force plate system showed no interaction effect (F(2,34) = 1.34; *p* = 0.29; *ηp*^2^ = 0.18) whilst the seven participants that performed the jump protocol with the ForceDecks force plate system demonstrated an interaction effect (F(2,34) = 3.93; *p* = 0.04; *ηp*^2^ = 0.28). However, post hoc analyses for the seven participants with the ForceDecks force plate system revealed no differences between time nor conditions (*p* > 0.05). The effect size calculations showed similar trends between the 11 participants (AMTI force plate system) and the seven participants (ForceDecks force plate system). Specifically, there was a greater decline in DJ-FT for the 11 participants (AMTI force plate system) with a large effect size at T24 (ES = 0.80) and moderate effect size at T48 (ES = 0.65) for the HYP modality than STR modality. However, the decline in DJ-FT for the seven participants (ForceDecks force plate system) was smaller at T24 (ES = 0.47), whilst larger at T48 (ES = 0.95) for the HYP modality than the STR modality.

### 3.5. Field-Specific Measures

The 10–20 m flying start, 10–40 m flying start and 20–40 m flying start were normally distributed. However, the rest of the field-performance measures were log-transformed based on normality assumptions. There was an interaction effect for the 0–10 m sprint time (F(2,34) = 4.12; *p* = 0.045; *ηp*^2^ = 0.20). Post hoc analyses showed significantly slower 0–10 m sprint time for the hypertrophy modality than strength modality at T48 (*p* = 0.03), although no differences were found between modalities for the other time points (*p* > 0.05). Furthermore, the 0–10 m sprint time was significantly slower at T48 compared to baseline (*p* = 0.032) during the hypertrophy modality, although there were no differences between baseline and T24 (*p* = 0.47). In addition, there were no differences between time points during the strength modality (*p* > 0.05). No interaction effects were evident for any of the other field-specific performance measures nor was there any main effect of time (Table 3). There were main effects of modality with 0–20 m (3.07 ± 0.14 vs. 3.12 ± 0.20 s, (F(1,17) = 5.14; *p* = 0.037; *ηp*^2^ = 0.23)) and 10–20 m (1.30 ± 0.08 vs. 1.33 ± 0.10 s, *p* = 0.040), with sprint times significantly faster for the strength compared to the hypertrophy modality. Effect size calculations showed slower sprint times for most measures (0–10 m sprint, 0–20 m sprint, 0–40 m sprint, 10–20 m flying start and 10–40 m flying start) during the hypertrophy modality than the strength modality at T24 with moderate-to-large effects. This effect size trend was also evident at T48, although for fewer sprint measures (0–10 m sprint and 0–20 m sprint). Effect size calculations also showed slower agility times for most measures (best time, average time and total time) and larger average RPE during the hypertrophy modality than strength modality at T24 with moderate effects. This effect size trend was also evident at T48, although for fewer agility times (average time and total time).

## 4. Discussion

According to the current findings, the athletes appeared to exhibit a greater level of EIMD (i.e., via indirect markers such as CK and DOMS), with moderate-to-large ES, when athletes were exposed to a hypertrophy resistance exercise session after several weeks of maximal strength training. This greater amount of muscle damage was accompanied by reduced explosive-strength (e.g., DJ-FT) and field-specific performance (e.g., slower 10 m, 20 m, 10–20 m flying start and agility performance). According to these results, if resistance training workload volume was acutely increased for athletes who have completed several weeks of heavy-loaded resistance training, a greater recovery period may be required following the initial resistance training session, particularly for field-specific conditioning sessions involving sprinting and agility activities.

Unfamiliar resistance exercise augments the level of DOMS and CK for several hours to days post-exercise, indicating the presence of EIMD [20]. The current study confirms previous findings with athletes exhibiting higher levels of CK and DOMS scores once exposed to an unaccustomed hypertrophy resistance exercise session, with moderate-to-large ES. In addition, there was a significant increase in muscular strength measures based on RM testing after the heavy-loaded mesocycle, as well as lower RPE measures during the heavy strength exercise session compared to the hypertrophy session. These findings confirm that the athletes adapted to the heavy-loaded resistance training exercises and perceived that the unaccustomed hypertrophy resistance exercise session was more strenuous. Thus, the lower level of EIMD evident following the strength session is likely due to the RBE [20]. Whilst there is evidence to suggest that it takes up to 6 months for individuals to return to pre-exercise conditions once exposed to muscle-damaging exercises, the degree of EIMD may be dependent upon the extent of change in training stimuli for resistance trained athletes [19]. The findings from the current study partly supported prior results where an acute increase in workload volume increased the level of EIMD for athletes [19]. Simmons, Doma [19], also reported that a reduction in acute workload volume lessened the level of EIMD, further confirming the link between training load and the level of muscle damage. Based on this relationship, it is plausible that the level of EIMD may be reduced when transitioning from higher workload volume to lower workload volume mesocycles during a linear resistance training periodization program. Further research will confirm this potential trend in a structured training context.

Given the physical demands involved in rugby league, in particular the high-intensity efforts, upper- and lower-body explosive-strength measures are a fundamental tool for fatigue monitoring assessment [3]. The current study demonstrated that athletes produced less lower-body explosive-strength (i.e., DJ-FT) following the hypertrophy session compared to the strength session, with moderate-to-large ES. The reduction in DJ-FT occurred possibly because the athletes were experiencing significant EIMD (i.e., CK, DOMS) due to the unaccustomed greater workload volume during the hypertrophy modality. Interestingly, upper-body explosive-strength measures were similar between hypertrophy and strength modalities. This lack of change in upper-body explosive-strength, despite impairment in lower-body explosive-strength, may be due to differences in the number of exercises for lower-body push to upper-body push movements within each exercise session. For example, there were four push movements for the lower-body, while only two push exercises were prescribed for the upper-body during each exercise session. This lower focus on upper-body movements resulted in less DOMS-PU and likely EIMD compared to the lower-body assessments (i.e., DOMS-SQ). The differences in workload volume between upper- and lower-body exercises is commonly prescribed by coaches, given the larger volume of muscles in the lower-body [20]. Therefore, athletes may not be impacted by increased resistance training workload volume for upper-body explosive-strength based on common training practices, suggesting less recovery may be required for upper-body conditioning exercises. Whilst the ES calculations exhibited similar trends for each force plate system, caution should be taken, given that the ANOVA results were distinct for each sub-group analyses.

Slower sprint performances (10 m, 20 m and 10–20 m flying start) during the hypertrophy modality were accompanied by greater levels of CK and DOMS in the current study and supported previous findings of impaired sprint performance during periods of EIMD [23]. Previously, it was speculated that sprint performance was impaired for several days following resistance training due to attenuation in muscle force generation capabilities with higher EIMD levels [23]. Our finding of a reduction in DJ-FT supports this reduced muscle force generation for athletes. Consequently, the quality of sprint-specific conditioning sessions may be compromised for several days when athletes who have completed several weeks of heavy-loaded resistance training focused on developing maximum strength are exposed to a hypertrophy resistance exercise session. Impaired sprint performance may also be due to altered sprint biomechanics [28] that may increase the risks of injuries. Further research examining the sprint biomechanics of competitive team sport athletes during the strength to hypertrophy transition may clarify the mechanism for EIMD-induced poorer performance.

Despite increased EIMD markers in our study with a concomitant reduction in lower-body explosive-strength and sprint performance, no statistically significant differences in time-to-completion were found within the repeated T-test protocol. Previous studies have also reported no changes in COD performance despite increased levels of EIMD following resistance and sport-specific training in elite volleyball [29] and basketball [30] players. However, ES calculations in the present study suggested small-to-moderate magnitude differences between modalities, indicating that repeated COD performance may have been modestly affected despite the absence of statistical significance. These findings therefore partly support those of Doma, Burt [23], who speculated that athletes may maintain the fastest completion time of a repeated COD protocol due to pacing strategies, even when average performance measures are impaired following resistance exercise. Therefore, it is probable that the athletes in the current study paced themselves during the repeated COD protocols, thereby diluting potential differences caused by EIMD symptoms. Consequently, whilst repeated COD performance appeared relatively preserved, modest impairments cannot be completely ruled out when athletes are exposed to increases in resistance training workload volume.

The current study identified the acute responses of a typical hypertrophy-style resistance training session in youth rugby league players who completed several weeks of heavy loaded resistance training focused on developing maximal strength. However, some limitations of the study should be acknowledged. This study examined several physical performance testing measures only, with investigation of more skilled performances, such as passing, kicking accuracy, catch and tackle technique, recommended for the future. Understanding how alteration of resistance training workload volume impacts upon an athlete’s ability to perform rugby league skills may provide further information for coaches to integrate physical and skills sessions during the same training program. Furthermore, it is possible that the level of EIMD may have varied between individuals due to varying training and playing experience in rugby league. Whilst we incorporated strict inclusion criteria with a minimum of two years of resistance training experience and a minimum of four years of playing rugby league, future research could consider obtaining such information to determine the association between training and playing experience with EIMD markers, given that previous exercise exposure may influence the level of EIMD as per the repeated bout effect. Whilst the athletes undertook maximal strength training during the initial four weeks, with no other forms of resistance training performed during this period, we cannot guarantee that prior exposure to hypertrophy resistance training had not influenced the acute responses to hypertrophy resistance training observed in this study. Furthermore, given that this study employed a more linear periodization approach during the four weeks of heavy-loaded mesocycle, the findings may not be extrapolated to an undulated periodization program, where the workload volume fluctuates within each training week. In addition, data were amalgamated from two separate force plate systems (AMTI and ForceDecks) due to technical difficulties. According to the sensitivity analyses, the findings could not be replicated when the data were analyzed separately for each force plate system, which may be attributable to differences in the engineering characteristics of the systems or the reduced statistical power resulting from dividing the sample into two groups. Whilst similar trends were reported with ES calculations for both force plate systems, caution should be exercised when interpreting the DJ-FT results, particularly given that an interaction effect was observed when analyzing the full sample. A further limitation of the present study is that the experimental design captured responses within a relatively narrow temporal window, focusing on the acute effects of a single hypertrophy-style resistance exercise session following a mesocycle of heavy-loaded resistance training. While this approach provides insight into the immediate responses to a sudden increase in workload volume, it may not fully reflect long-term periodization practices, where multiple hypertrophy sessions are typically performed and athletes may adapt rapidly through the repeated-bout effect. Consequently, the observed elevations in markers of exercise-induced muscle damage and the small reductions in selected performance measures should be interpreted within the context of these acute responses and may not necessarily reflect the responses that occur with continued exposure to hypertrophy-oriented resistance training. In addition, we were unable to blind the assessors for DOMS reporting, which may have introduced reporting bias. However, it is important to note that the acute intervention sessions (i.e., STR and HYP modalities) were undertaken in sequence, rather than randomized, and the assessors were highly experienced in employing the DOMS scale. Finally, this study examined the exposure to only one hypertrophy resistance training session after the completion of several weeks of heavy-loaded resistance training. Future research is recommended to investigate how the athletes perform throughout the entire hypertrophy mesocycle, which may clarify the role of the RBE and EIMD on athletes’ training and performance.

## 5. Conclusions

In conclusion, the level of EIMD was augmented when athletes completed a hypertrophy-style resistance exercise session following a mesocycle of heavy-loaded resistance training focused on developing maximal strength, possibly due to a greater acute workload volume. This was accompanied by moderate reductions in sprint and explosive-strength performance, which may influence subsequent explosive-strength and field-based training sessions when athletes are exposed to an increase in resistance training workload volume. However, caution should be taken when interpreting the explosive strength measures, as two separate force plate systems were used for analyses. Coaches could monitor the level of EIMD for athletes that complete a mesocycle of heavy-loaded resistance training and are periodized to undertake their initial resistance exercise session with increased workload volume. If a high level of EIMD is detected, greater recovery may be considered, particularly if implementing field-specific conditioning sessions to help maintain training quality. Future longitudinal research examining repeated exposure to hypertrophy-oriented resistance training is warranted to better understand how these acute responses evolve over time.

## Figures and Tables

**Table 1:** Resistance training program across the 6 weeks.

	Week 1	Week 2	Week 3	Week 4 *	Week 5 **	Week 6 ***
1a—Barbell back squat	4 × 3–5 @ 70–90% RM	4 × 3 @ 70–92% RM	4 × 2–3 @ 70–95% RM	4 × 1–3 @ 70–100% RM	4 × 1–3 @ 70–95% RM	3 × 8–10 @ 65–70% RM
1b—Single leg calf raise	4 × 5 @ 80–85% RM	4 × 5 @ 85% RM	4 × 5 @ 85–88% RM	4 × 5 @ 85–90% RM	4 × 5 @ 80–85% RM	3 × 12 @ 60% RM
2a—Barbell bench press	4 × 3–5 @ 70–90% RM	4 × 3 @ 70–92% RM	4 × 2–3 @ 70–95% RM	4 × 1–3 @ 70–100% RM	4 × 1–3 @ 70–95% RM	3 × 8–10 @ 65–70% RM
2b—Supinated chin ups	4 × 4–5 @ 60–65% RM	4 × 3–4 @ 65–70% RM	4 × 2–3 @ 70–85% RM	4 × 1–3 @ 70–100% RM	4 × 1–3 @ 75–95% RM	3 × 8 @ 35% RM
3a—Hex bar deadlift	4 × 3–5 @ 70–90% RM	4 × 3 @ 70–92% RM	4 × 2–3 @ 70–95% RM	4 × 1–3 @ 70–100% RM	4 × 1–3 @ 70–95% RM	3 × 8–10 @ 65–70% RM
3b—Barbell split squat	4 × 3–5 @ 75–80% RM	4 × 3 @ 80–90% RM	4 × 3 @ 80–90% RM	4 × 3 @ 80–95% RM	4 × 3 @ 75–90% RM	3 × 8 @ 50% RM
4a—Barbell bench row	4 × 4–5 @ 80–85% RM	4 × 3–4 @ 80–88% RM	4 × 3–4 @ 80–90% RM	4 × 3 @ 80–95% RM	4 × 3 @ 80–90% RM	3 × 8–10 @ 65–70% RM
4b—Seated dumbbell shoulder press	4 × 5 @ 75–80% RM	4 × 3–5 @ 80–85% RM	4 × 3 @ 80–90% RM	4 × 3 @ 80–95% RM	4 × 3 @ 80–90% RM	3 × 8–10 @ 65–70% RM

Values denote sets x repetitions; * = strength re-test; ** = strength modality week; *** = hypertrophy modality week; RM = repetition maximum; ST = strength; HYP = hypertrophy. Exercises were performed as a superset with differing letters denoting paired exercises, for example, 1a and 1b were completed as a superset.

**Table 2:** Muscle damage marker and explosive strength measures following strength and hypertrophy exercise sessions for youth rugby league players.

	Baseline	T24 (%Δ)	T48 (%Δ)
DOMS-SQ (AU)			
STR	1.78 ± 0.8	3.22 ± 1.1 *,† (123.1 ± 327.8)	2.83 ± 0.8 *,† (87.0 ± 87.7)
HYP	1.61 ± 0.6	6.00 ± 1.6 † (327.8 ± 204.5)	4.56 ± 1.3 † (220.4 ± 146.6)
ES for STR vs. HYP (95% CI)		−1.0 (−1.8, −0.6) ‡	−0.97 (−1.7, −0.5) ‡
DOMS-PU (AU)			
STR	1.6 ± 0.8	1.9 ± 0.6 * (50.9 ± 70.6)	1.7 ± 0.5 * (35.2 ± 63.1)
HYP	1.5 ± 0.6	4.2 ± 1.5 † (239.8 ± 195.0)	3.2 ± 1.0 † (143.5 ± 124.3)
ES for STR vs. HYP (95% CI)		−1.12 (−2.0, −0.5) ‡	−1.0 (−1.8, −0.4) ‡
Creatine kinase (U∙L^−1^)			
STR	185 ± 114	360 ± 429 (83.2 ± 92.7)	322 ± 232 (90.4 ± 117.0)
HYP	192 ± 102	651 ± 554 (306.1 ± 326.3)	481 ± 433 (216.4 ± 301.2)
ES for STR vs. HYP (95% CI)		−0.6 (−1.5, −0.3) §	−0.5 (−1.1, 0.05)
Drop jump contact time (sec)			
STR	0.38 ± 0.11	0.39 ± 0.11 (5.5 ± 23.7)	0.35 ± 0.08 (−4.3 ± 20.3)
HYP	0.37 ± 0.10	0.39 ± 0.11 (8.6 ± 23.5)	0.39 ± 0.13 (8.4 ± 34.2)
ES for STR vs. HYP (95% CI)		−0.1 (−0.4, 0.2)	−0.4 (−1.0, 0.1)
Drop jump flight time (sec)			
STR	0.60 ± 0.06	0.60 ± 0.05 * (−0.6 ± 3.4)	0.61 ± 0.04 * (1.2 ± 8.6)
HYP	0.60 ± 0.06	0.58 ± 0.05 † (−3.6 ± 4.6)	0.58 ± 0.05 † (−4.3 ± 4.5)
ES for STR vs. HYP (95% CI)		0.7 (0.1, 1.4) §	0.7 (0.2, 1.4) §
Drop jump RSI			
STR	1.75 ± 0.55	1.63 ± 0.53 (−5.1 ± 14.3)	1.73 ± 0.61 (5.5 ± 39.5)
HYP	1.78 ± 0.51	1.66 ± 0.52 (−5.6 ± 15.8)	1.72 ± 0.67 (−2.4 ± 23.5)
ES for STR vs. HYP (95% CI)		0.03 (−0.4, 0.5)	0.2 (−0.3, 0.8)
Plyometric push up flight time (sec)			
STR	0.42 ± 0.13	0.44 ± 0.12 (7.1 ± 1.5)	0.46 ± 0.11 (15.2 ± 25.5)
HYP	0.42 ± 0.15	0.41 ± 0.14 (0.3 ± 18.8)	0.44 ± 0.14 (5.6 ± 23.0)
ES for STR vs. HYP (95% CI)		0.4 (−0.01, 0.8)	0.4 (−0.3, 1.0)

STR = strength modality; HYP = hypertrophy modality; * = *p* < 0.05 vs. HYP; † = *p* < 0.05 vs. baseline within same exercise session; ‡ = large effect size (>0.8); § = moderate effect size (>0.5); %Δ = percentage change from baseline; ES = effect size calculation between the strength and hypertrophy modality at T24 and T48; DOMS-SQ = delayed onset of muscle soreness during a squat; DOMS-PU = delayed onset of muscle soreness during a push up; AU = arbitrary unit; U∙L^−1^ = units per liter; RSI = reactive strength index; sec = seconds; T24 = 24 h after resistance exercise session; T48 = 48 h after resistance exercise session.

**Table 3:** Field-specific performance measures following strength and hypertrophy exercise sessions for youth rugby league players.

	Baseline	T24 (%Δ)	T48 (%Δ)
0–10 m sprint (sec)			
STR	1.77 ± 0.08	1.78 ± 0.10 (0.9 ± 2.8)	1.76 ± 0.06 (−0.3 ± 4.1)
HYP	1.77 ± 0.08	1.80 ± 0.11 (1.5 ± 3.1)	1.82 ± 0.11 (2.6 ± 3.8)
ES for STR vs. HYP (95% CI)		−0.2 (−0.6, 0.2)	−0.7 (−1.4, −0.04) §
0–20 m sprint (sec)			
STR	3.08 ± 0.19	3.08 ± 0.18 (0.3 ± 2.0)	3.05 ± 0.11 (−0.6 ± 4.6)
HYP	3.09 ± 0.17	3.13 ± 0.20 (1.7 ± 2.1)	3.14 ± 0.22 (1.7 ± 2.7)
ES for STR vs. HYP (95% CI)		−0.6 (−1.2, −0.08) §	−0.6 (−1.2, 0.001) §
0–40 m sprint (sec)			
STR	5.50 ± 0.45	5.57 ± 0.42 (0.4 ± 1.6)	5.50 ± 0.24 (−0.6 ± 5.8)
HYP	5.57 ± 0.43	5.67 ± 0.47 (1.7 ± 2.1)	5.64 ± 0.50 (1.2 ± 2.3)
ES for STR vs. HYP (95% CI)		−0.7 (−1.4, −0.02) §	−0.4 (−1.0, 0.2)
10–20 m flying start (sec)			
STR	1.31 ± 0.11	1.30 ± 0.09 (−0.4 ± 2.9)	1.30 ± 0.06 (−0.9 ± 5.7)
HYP	1.32 ± 0.10	1.34 ± 0.10 (1.9 ± 2.4)	1.32 ± 0.11 (0.6 ± 2.8)
ES for STR vs. HYP (95% CI)		−0.9 (−1.6, −0.2) ‡	−0.3 (−0.96, 0.3)
10–40 m flying start (sec)			
STR	3.78 ± 0.38	3.79 ± 0.33 (0.2 ± 2.0)	3.74 ± 0.19 (−0.6 ± 6.7)
HYP	3.80 ± 0.36	3.87 ± 0.37 (1.8 ± 2.2)	3.82 ± 0.39 (0.6 ± 2.4)
ES for STR vs. HYP (95% CI)		−0.7 (−1.4, −0.1) §	−0.5 (−1.1, 0.04)
20–40 m flying start (sec)			
STR	2.47 ± 0.27	2.48 ± 0.25 (0.5 ± 2.5)	2.45 ± 0.13 (−0.5 ± 7.3)
HYP	2.48 ± 0.27	2.53 ± 0.27 (1.7 ± 2.8)	2.50 ± 0.28 (0.6 ± 2.6)
ES for STR vs. HYP (95% CI)		−0.4 (−1.1, 0.21)	−0.2 (−0.8, 0.4)
Agility T-test best time (sec)			
STR	9.95 ± 0.57	9.89 ± 0.73 (−0.6 ± 3.8)	9.73 ± 0.56 (−2.0 ± 5.6)
HYP	9.81 ± 0.68	9.96 ± 0.78 (1.5 ± 2.7)	9.81 ± 0.58 (0.1 ± 4.7)
ES for STR vs. HYP (95% CI)		−0.6 (−1.26, 0.02) §	−0.39 (−0.9, 0.1)
Agility T-test average time (sec)			
STR	10.25 ± 0.62	10.14 ± 0.76 (−1.1 ± 3.3)	10.03 ± 0.53 (−2.0 ± 5.1)
HYP	10.14 ± 0.71	10.23 ± 0.81 (0.9 ± 2.8)	10.18 ± 0.67 (0.5 ± 2.3)
ES for STR vs. HYP (95% CI)		−0.6 (−1.3, −0.01) §	−0.6 (−1.2, −0.01) §
Agility T-test total time (sec)			
STR	61.51 ± 3.73	60.84 ± 4.57 (−1.1 ± 3.3)	60.17 ± 3.19 (−2.0 ± 5.2)
HYP	60.85 ± 4.27	61.42 ± 4.87 (0.9 ± 2.8)	61.11 ± 4.02 (0.5 ± 2.3)
ES for STR vs. HYP (95% CI)		−0.6 (−1.3, −0.02) §	−0.6 (−1.2, 0.01) §
Agility T-test fatigue index (%)			
STR	−3.08 ± 1.83	−2.55 ± 2.10 (17.1 ± 112.7)	−3.10 ± 1.40 (37.9 ± 94.6)
HYP	−3.37 ± 2.16	−2.79 ± 1.32 (19.1 ± 103.3)	−3.88 ± 3.34 (57.0 ± 165.0)
ES for STR vs. HYP (95% CI)		−0.02 (−0.5, 0.4)	−0.1 (−0.6, 0.3)
Agility T-test average RPE (AU)			
STR	5.8 ± 1.0	5.6 ± 0.7 (−2.7 ± 11.5)	5.6 ± 0.7 (−3.6 ± 12.3)
HYP	5.5 ± 0.7	5.9 ± 0.7 (7.1 ± 16.0)	5.7 ± 0.7 (2.7 ± 12.9)
ES for STR vs. HYP (95% CI)		−0.7 (−1.3, −0.09) §	−0.5 (−1.1, 0.2)
Agility T-test peak RPE (AU)			
STR	7.6 ± 1.2	7.3 ± 1.0 (−2.7 ± 12.0)	7.6 ± 0.6 (2.8 ± 14.2)
HYP	7.6 ± 1.0	7.9 ± 0.8 (5.4 ± 16.8)	7.9 ± 1.0 (4.6 ± 15.7)
ES for STR vs. HYP (95% CI)		−0.5 (−1.2, 0.1)	−0.1 (−0.6, 0.4)

STR = strength modality; HYP = hypertrophy modality; ‡ = large effect size (>0.8); § = moderate effect size (>0.5); %Δ = percentage change from baseline; ES = effect size calculation between the strength and hypertrophy modality at T24 and T48; RPE = rating of perceived exertion; sec = seconds; AU = arbitrary units; T24 = 24 h after resistance exercise session; T48 = 48 h after resistance exercise session.

## Data Availability

Data is unavailable due to privacy restrictions.

## Supplementary Materials

The following supporting information can be downloaded at: https://www.mdpi.com/article/10.3390/sports14040142/s1, Figure S1: Spaghetti plot of individual data points for creatine kinas.

## Abbreviations

The following abbreviations are used in this manuscript:

RM	Repetition maximum
EIMD	Exercise-induced muscle damage
DOMS	Delayed onset of muscle soreness
CK	Creatine kinase
STR	Maximal strength exercise session
HYP	Hypertrophy exercise session
T24	Time point at 24 h after STR and HYPs
T48	Time point at 48 h after STR and HYPs
DOMS-SQ	Delayed onset of muscle soreness during a squat maneuver
DOMS-PU	Delayed onset of muscle soreness during a push-up maneuver
RPE	Rating of perceived exertion
DJ-FT	Drop jump flight time
RSI	Reactive strength index
PP-FT	Plyometric push-up flight time
