# Supplementary figures and images for: The Acute Effect of Increasing Resistance Training Workload Volume on Muscle Damage Markers and Performance in Heavy Resistance-Trained Youth Athletes
Source: Sports (Basel). 2026 Apr 3;14(4):142. doi: 10.3390/sports14040142 (PMC13119994; doi:10.3390/sports14040142)

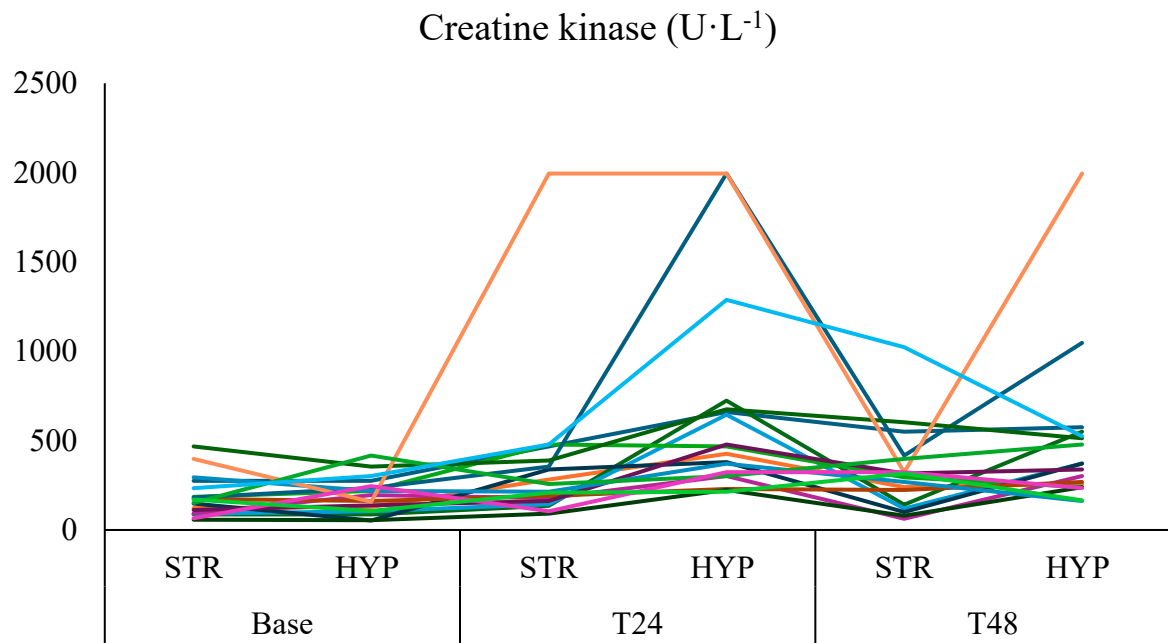

**Figure S1:** Spaghetti plot of individual data points for creatine kinas.

Supplement: Supplementary file 1 [file sports-14-00142-s001.zip › sports-4126722-supplementary.pdf]
